# Management of infected nasal dermoid cysts and sinuses

**DOI:** 10.1093/jscr/rjab041

**Published:** 2021-04-06

**Authors:** Ryan Bishop, Cameron Sheehan, Patrick Walz, Charlemagne Kern, Charles Elmaraghy

**Affiliations:** The Ohio State University College of Medicine, Columbus, OH, USA; The Ohio State University College of Medicine, Columbus, OH, USA; The Ohio State University College of Medicine, Columbus, OH, USA; Department of Pediatric Otolaryngology, Nationwide Children’s Hospital, Columbus, OH, USA; Department of Pediatric Otolaryngology, Nationwide Children’s Hospital, Columbus, OH, USA; The Ohio State University College of Medicine, Columbus, OH, USA; Department of Pediatric Otolaryngology, Nationwide Children’s Hospital, Columbus, OH, USA

## Abstract

This study investigates outcomes of surgical management of pediatric patients with nasal dermoids with prior infection. A retrospective review at Nationwide Children’s Hospital, a large free-standing pediatric hospital in the Midwestern USA, was performed. Patients were identified by the Current Procedural Terminology codes 30124 (simple excision of dermoid cyst) and 30125 (complex excision of nasal dermoid cyst) from 2011 to 2016. Demographic, imaging data, surgical findings, microbiological data and recurrence rates were collected for these patients. Descriptive statistical investigation was performed. In total, 14 patients were identified, 4 of the 14 patients (28.5%) had recurrent infection and required additional surgery. Three of seven patients required incision and drainage prior to definitive excision. One of seven patients in the infected group had recurrence. Prior infection does not increase the recurrence rate and almost half of the patients required I&D prior to definitive management.

## INTRODUCTION

**Figure 1:**
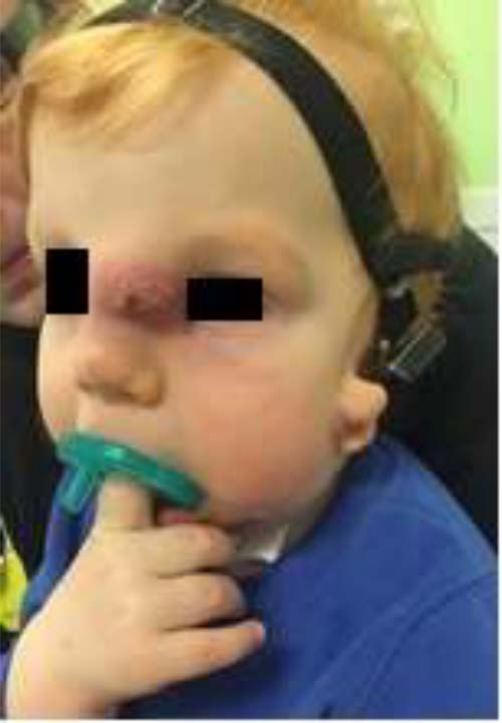
Presurgical appearance of an infected nasal dermoid cyst.

Nasal dermoid cysts and sinuses are a rare occurrence in children with presentation in one out of 20 000–40 000 births [1]. Like other dermoid cysts, their embryologic origins are derived from the ectoderm and mesoderm [2]. Because of this developmental lineage, these cysts are comprised of a stratified squamous epithelium and could contain more adnexal structures such as hair follicles. Dermoid cysts can be associated with midline defects and Gorlin syndrome; otherwise, they typically occur in isolation. The usual clinical presentation involves a child with a midline mass or swelling at the nasal dorsum associated with an infection of the nasal dermoid cyst (Fig. 1). Other possible diagnoses to consider are gliomas, polyps or encephalocoeles [3]. Bradley and colleagues established that nasal dermoid cysts should be qualified by ‘simple’ or ‘complex’ depending on the surgical extent of the tissues involved [4].

Magnetic resonance imaging (MRI) or computerized tomography (CT) are the most common imagine modalities. Winterton and colleagues determined that the positive and negative predictive values for intracranial extension were 85.7% and 50% for CT and 100% and 50% for MRI, respectively [5].

Urgent need for surgical intervention develops when these cysts become infected. Morrissey and colleagues described an alternative to the standard vertical midline incision; they performed an external rhinoplasty approach for a series of dermoid cysts. They found their exposure had improved and the cosmetic result, especially in children, was superior [6].

There remains a paucity of literature on this particularly rare manifestation of dermoid cyst. With its potential for serious complication and infection, this surgical problem and potential treatment modalities should be explored though the aggregation of patient-level data.

## METHODS

A retrospective review at Nationwide Children’s Hospital, a large free-standing pediatric hospital in the Midwestern USA, was performed. Patients were identified by the Current Procedural Terminology codes 30124 (simple excision of dermoid cyst) and 30125 (complex excision of nasal dermoid cyst) from 2011 to 2016. Demographics, imaging data, surgical findings, microbiological data, recurrence rates were collected for these patients. Descriptive statistical investigation was performed.

## RESULTS

**Figure 2:**
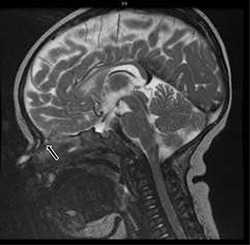
T2-weighted sagittal MRI of hyperintense soft tissue findings in the setting of a nasal dermoid cyst penetrating the anterior cranial fossa.

**Figure 3:**
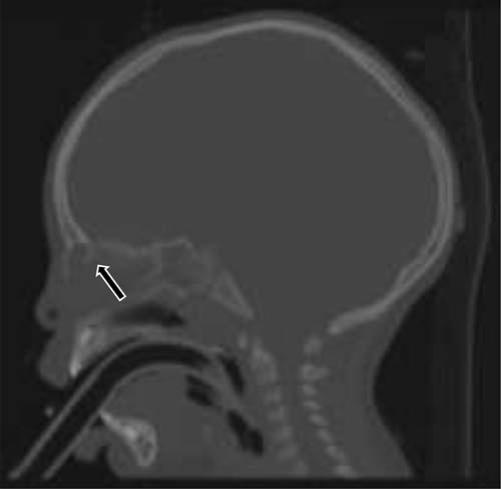
Sagittal cranial CT scan with bone window showing splaying of the nasal bone; a severe osseous defect potentiating extension into the intracranial space.

A total of fourteen patients were identified. The age range of these patients was 6 months to 17 years. There was an equal gender distribution. An average of 11 weeks lapsed between initial presentation and definitive excision. All patients underwent MRI; all patients exhibited an abnormality, most commonly isointense T1 and hyperintense T2 soft tissue findings in the area of the nasal dorsum/glabella (Fig. 2). Eleven of the fourteen patients (78.6%) underwent a CT. Of those eleven patients, two patients exhibited a splaying of nasal bones (Fig. 3). Fifty percent of the patients required preoperative antibiotics for infected nasal dermoid cysts; one patient was admitted to the infectious disease service for intravenous antibiotic therapy prior to formal surgical excision. One of the fourteen patients (7.1%) had a formal excision with a rhinoplasty approach, while the majority thirteen of the fourteen patients (92.8%) underwent an open vertical incision for the surgical approach. Four of the fourteen patients (28.5%) had recurrent infection and required additional surgery. Three of seven patients required incision and drainage prior to definitive excision. One of seven patients in the infected group had recurrence. Three of the seven patients in the non-infected group recurred. Bacterial cultures in all of these patients did not identify common pathogens. Surgical pathology revealed acute on chronic inflammation in 50% of cases; three of the fourteen patients (21.4%) revealed multinucleated giant cells on microscopic analysis. Two of the fourteen total patients had intracranial extension. None of these patients developed meningitis.

## DISCUSSION

This case series represents a single-institution retrospective review at Nationwide Children’s Hospital, a large free-standing pediatric hospital in the Midwestern USA. These data describe a rare surgical problem. In the two cases with intracranial extension described, neurosurgical expertise was present in the operating room to perform a complete excision. The studies previously published recommend this multi-disciplinary approach [2, 3, 5, 7]. Winterton and colleagues report that this multidisciplinary approach, even when intracranial involvement is not completely evident but cannot be ruled out, can achieve lower recurrence rates and minimize the morbidity and the potential for serious meningeal infections [5].

Surgical technique has often been considered as a driver for outcomes as well. Moses and colleagues reported a 15-year protocol-driven experience in which no recurrences were reported from their cohort of patients that underwent open excision of their simple dermoid cysts. Of those in their cohort, which were found to have intracranial extension on MRI, one patient did have a recurrence that required subsequent treatment [1]. Re and colleagues described two patients who underwent endonasal endoscopic approach to treat intracranial nasal dermoid sinus cysts in children with success [8].

Although cases of meningitis and other serious findings such as periorbital involvement, extension of cellulitis, and osteomyelitis are reported in the literature, we did not appreciate this in our cohort of pediatric patients [9]. A search of the current literature revealed little about the bacterial organisms specifically suspected of playing a role in the development of infected dermoid cysts.

The study has the expected limitations of this particular study design. The retrospective nature of this review relies on the accuracy of these data as they are extracted from the electronic medical record. Our sample size is also small; however, our focus was on patients that were appropriately captured for their specific diagnosis of nasal dermoid cyst, proper preoperative imaging, operative findings and postoperative outcomes.

## CONCLUSIONS

Overall, more than half of the patients with infected nasal dermoid required admission as well as incision and drainage prior to definitive surgery. In this case series, the recurrence rate of patients with infections was lower than in those without infection. No cases of meningitis resulted from infected nasal dermoid cysts. In summary, we recommend imaging with high resolution axial and coronal CT and/or MRI. In the case of infected nasal dermoid cysts, incision and drainage may be necessary prior to surgery, and appropriate pre-operative antibiotics should be employed. With regard to surgical management, we recommend direct midline approach for excision, specifically in the case of prior infection.

## CONFLICT OF INTEREST STATEMENT

The authors have no financial conflicts of interest to disclose.

## FUNDING

This project is supported by intramural funding from the Department of Otolaryngology at Nationwide Children’s Hospital and the Center for Surgical Outcomes Research at The Research Institute.
